# SARS-CoV-2 N protein antagonizes type I interferon signaling by suppressing phosphorylation and nuclear translocation of STAT1 and STAT2

**DOI:** 10.1038/s41421-020-00208-3

**Published:** 2020-09-15

**Authors:** Jingfang Mu, Yaohui Fang, Qi Yang, Ting Shu, An Wang, Muhan Huang, Liang Jin, Fei Deng, Yang Qiu, Xi Zhou

**Affiliations:** 1 State Key Laboratory of Virology, Wuhan Institute of Virology, Center for Biosafety Mega-Science, Chinese Academy of Sciences (CAS), Wuhan, Hubei 430071 China; 2 Joint Laboratory of Infectious Diseases and Health, Wuhan Institute of Virology & Wuhan Jinyintan Hospital, CAS, Wuhan, Hubei 430023 China; 3 The University of Chinese Academy of Sciences, Beijing 100049, China; 4 Institute of Microbiology, Jiangxi Academy of Sciences, Nanchang, Jiangxi 330029 China

**Keywords:** Immunology, Molecular biology

Dear Editor,

The outbreak of coronavirus disease 2019 (COVID-2019) has spread rapidly across the globe since late 2019 and has become a pandemic^1^. The causative pathogen of COVID-19 is a novel strain of coronavirus named as severe acute respiratory syndrome coronavirus 2 (SARS-CoV-2). SARS-CoV-2 is an enveloped virus with single-stranded, positive-sense RNA genome, which belongs to the genus *Betacoronavirus* of the family *Coronaviridae*, and is the third coronavirus reported to cause severe respiratory diseases, together with SARS-CoV and Middle East respiratory syndrome coronavirus (MERS-CoV). The genomic RNA of SARS-CoV-2 is ~30 kb that contains 12 open reading frames (ORFs), encoding structural proteins envelope (E), spike (S), membrane (M), and nucleocapsid (N).

Interferon (IFN)-mediated antiviral pathway is one of the major innate immune mechanisms against invading viral pathogens, which consists of two stages including IFN activation and IFN signaling. In the former one, viral infection rapidly induces expression and secretion of type I and III IFNs. In the latter stage, secreted type I and III IFNs bind to cell surface receptors to initiate the downstream Janus kinase (JAK)-signal transducer and activator of transcription (STAT) signaling. The binding of IFNs to their receptors activates and phosphorylates the intracellular tyrosine kinases including JAK1, JAK2, JAK3, and tyrosine kinase 2 (TYK2), resulting in the subsequent phosphorylation and activation of STAT1 and STAT2. Phosphorylated STAT1 and STAT2 can form heterodimers and interact with IFN regulatory factor 9 (IRF9) to form IFN-stimulated gene factor 3 (ISGF3) transcription complex, which translocate to the nucleus and binds to the IFN-stimulated response element (ISRE) of the IFN-stimulated gene (ISG) promoter, activating ISGs expression to establish host antiviral state^2^.

The type I interferon (IFN-I) immune signaling has been found to be dampened in COVID-19 cases, raising the hypothesis that SARS-CoV-2 uses some mechanism(s) to evade IFN-I-mediated antiviral innate immunity, which contributes to the pathogenicity of SARS-CoV-2. Indeed, during coevolution and arms race with host immune systems, many viruses have developed elaborate strategies to circumvent IFN signaling. However, it remains largely unanswered whether SARS-CoV-2 encodes any protein to counteract the IFN system.

We sought to explore whether SARS-CoV-2 N protein suppresses IFN signaling by examining the effect of N protein on Sendai virus (SeV)-induced ISRE-promoter activation via the luciferase reporter assay. We found that ectopic expression of N protein significantly inhibited the activation of ISRE activation induced by SeV infection (Fig. 1a), and real-time quantitative PCR (qRT-PCR) analysis also showed that SeV-induced expressions of ISG56, ISG54, and ISG15 were significantly inhibited in the presence of N protein (Fig. 1b and Supplementary Fig. S1a, b). Our further results showed that SARS-CoV-2 N protein also exhibited a significant inhibitory effect on ISRE-promoter activation induced by either IFN-2α or IFN-β (Fig. 1c, d), indicating that SARS-CoV-2 N protein can act as an antagonist of IFN signaling.

**Fig. 1:**
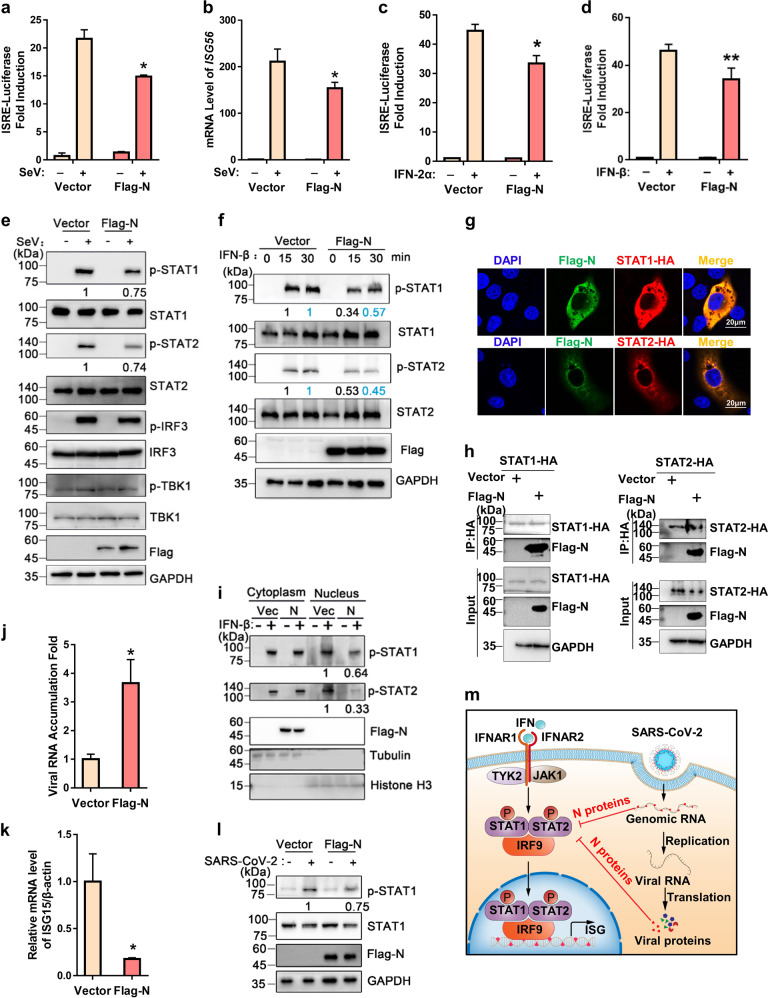
SARS-CoV-2 N protein antagonizes IFN signaling. **a**, **c**, **d** 293T cells were co-transfected with plasmids as indicated. At 24 h post transfection (hpt), cells were then infected with SeV (**a**), or treated with IFN-2α (**c**) or IFN-β (**d**), and at 12 hpi, the reporter activity was detected by dual luciferase reporter system. **b** 293T cells were transfected with the plasmid as indicated. At 24 hpt, cells were then infected with SeV. Total RNAs were extracted at 12 hpi. The mRNA levels of ISG56 were measured by qRT-PCR. **e** 293T cells were transfected with the plasmid as indicated. At 24 hpt, cells were then infected with SeV and cell lysates were harvested and analyzed by western blotting. **f** 293T cells were transfected with the plasmid as indicated. At 24 hpt, cells were then treated with or without IFN-β for 15 or 30 min. Cell lysates were harvested and analyzed by western blotting. **g** 293T cells were transfected with plasmids as indicated. At 24 hpt, cells were subjected to immunofluorescence staining with antibodies as indicated. Scale bars, 20 μm. **h** 293T cells were transfected with the plasmid as indicated. At 48 hpt, the cells were harvested and subjected to co-IP assay. **i** 293T cells were transfected with the plasmid as indicated. At 24 hpt, cells were then treated with or without IFN-β for 30 min. Cells were subjected to cell fractionation assay. **j**, **k** HepG2 cells were transfected with the plasmid as indicated. At 24 hpt, cells were infected with SARS-CoV-2 at a MOI of 0.1. At 24 hpi, total RNAs were extracted and the levels of SARS-CoV-2 RNA (**j**) and ISG15 (**k**) were measured by qRT-PCR analysis. **l** HepG2 cells were transfected with the plasmid as indicated. At 24 hpt, cells were infected with or without SARS-CoV-2 at a MOI of 0.1, and at 24 hpi, cell lysates were harvested and analyzed by western blotting. **m** Working model. SARS-CoV-2-encoded structural protein N interacts with STAT1 and STAT2, and suppresses the phosphorylation and nuclear translocation of STAT1 and STAT2, which blocks IFN signaling. Student’s *t*-test was used for estimation of statistical significance. *P < 0.05 and **P < 0.01. Data are shown as means ± SD from three independent experiments. The densities of blots were analyzed with ImageJ software.

To further investigate the detailed mechanism by which SARS-CoV-2 N suppresses IFN signaling, we examined the activation/expression of the components within JAK/STAT pathway in response to SeV infection. To this end, 293T cells transiently- or stably-expressing N protein were infected with SeV for 12 h and then analyzed via western blotting. As shown in Fig. 1e and Supplementary Fig. S1c, ectopic expression of SARS-CoV-2 N did not affect the steady-state levels of STAT1 and STAT2, while the phosphorylation of STAT1 and STAT2 (p-STAT1, at Tyr701 and p-STAT2, at Tyr690) were obviously reduced in cells expressing N protein compared to those in control cells. These results indicate that SARS-CoV-2 N protein can inhibit the phosphorylation of STAT1 and STAT2 induced by SeV infection. We also found that p-STAT1 and p-STAT2 were reduced in the presence of N protein at 15 min or 30 min post induction by IFN-β (Fig. 1f). Together, our findings indicate that SARS-CoV-2 N protein could antagonize IFN-I signaling via inhibiting the phosphorylation of STAT1 and STAT2.

We sought to examine if N protein can associate with STAT1 and STAT2 by examining their subcellular localization via confocal microscopy. Our results showed that N protein obviously co-localized with both STAT1 and STAT2 in cytoplasm (Fig. 1g). Moreover, Flag-tagged N protein could be co-immunoprecipitated (co-IP) together with HA-tagged STAT1 or STAT2 (Fig. 1h). These results show that SARS-CoV-2 N can interact with STAT1 and STAT2 in cells. Moreover, our result showed that SARS-CoV-2 N interfered with the interactions of STAT1 with JAK1 and STAT2 with TYK2 (Supplementary Fig. S1d, e), respectively, suggesting that SARS-CoV-2 N protein can competitively bind to STAT1/STAT2 with the downstream kinases and in turn inhibit their phosphorylation.

IFN-induced STAT1/STAT2 nuclear translocation is the essential step for the antiviral signal transduction. We next examined whether IFN-induced STAT1/STAT2 nuclear translocation can be affected by N protein. 293T cells expressing N protein were fractionated into cytoplasmic and nuclear fractions at 30 min post IFN-β induction. Our results showed that the levels of p-STAT1 and p-STAT2 were reduced in the nuclear fraction in the presence of N protein (Fig. 1i). Together, our findings indicate that SARS-CoV-2 N protein can interact with STAT1 and STAT2, and suppress their nuclear translocation induced by IFN.

Previous studies have shown that different functional domains of SARS-CoV N protein is critical for the IFN-I antagonizing^3^. We constructed the vectors for the residues 1-361 (N^1–361^) and 362-422 (N^362–422^) of SARS-CoV-2 N and examined their effects on ISRE-promoter activation induced by SeV in 293T cells. We found that the N-terminal 1-361 amino acids of SARS-CoV-2 N was sufficient to suppress the activation of ISRE-promoter (Supplementary Fig. S2a). Consistently, the levels of p-STAT1 were obviously reduced in the presence of N^1–361^ or full-length N protein, whereas N^362–422^ showed minimal effect on phosphorylation of STAT1 (Supplementary Fig. S2b). In addition, confocal microscopy analyses showed that similar with full-length N protein, N^1–361^ also co-localized with STAT1 and STAT2 in cytoplasm, whereas N^362–422^ lost the co-localization with STAT1 or STAT2 (Supplementary Fig. S2c, d). Moreover, co-IP assays also confirmed that N^1–361^ interacted with STAT1 and STAT2 in cells, while N^362–422^ failed to do so (Supplementary Fig. S2e, f). Collectively, these results indicate that the N-terminal 1-361 amino acids of SARS-CoV-2 N protein is sufficient for its IFN-I antagonizing activity. Moreover, to localize the key amino acid residues for N protein function, we aligned the amino acid sequences of N proteins of SARS-CoV, MERS-CoV, and SARS-CoV-2 (Supplementary Fig. S3), and then constructed a series of truncations based on the conserved motifs (Supplementary Fig. S4a). The protein-protein interactions of these mutants with STAT1 and STAT2 were examined via co-IP assays. Our data showed that deletion of amino acids 319-422 (Flag-N^Δ^^319–422^) resulted in a complete loss of interaction between N protein and HA-STAT1/STAT2 (Supplementary Fig. S4b, c), indicating that C-terminal 319-422 amino acids of SARS-CoV-2 N protein is indispensable for its binding to STAT1 and STAT2.

After determining that N protein of SARS-CoV-2 contains the activity to antagonize IFN-I signaling, we sought to examine whether it plays a role in SARS-CoV-2 infection. To this end, HepG2 cells expressing N protein or empty vector were infected with SARS-CoV-2 at MOI of 0.1, and the viral RNA accumulation in cells were determined at 24 h post infection (hpi). Our results showed that viral RNA replication was significantly elevated in SARS-CoV-2-infected cells in the presence of N overexpression (Fig. 1j). Consistently, the mRNA levels of ISG15 were also reduced in SARS-CoV-2-infected cells with N overexpression (Fig. 1k and Supplementary Fig. S5). Moreover, ectopic expression of SARS-CoV-2 N inhibited the phosphorylation of STAT1 in the context of SARS-CoV-2 infection (Fig. 1l). Taken together, our results showed that overexpression of N protein could efficiently enhance the replication of SARS-CoV-2 via antagonizing IFN-I signaling in infected human cells.

Coronavirus N protein is a multifunctional viral protein involved in the processes such as viral assembly, budding, RNA replication, and host cellular response^4^. Moreover, N proteins of SARS-CoV-2 and SARS-CoV have been found to antagonize antiviral RNA interference response in human cells via their RNA-binding activities^5^. Our findings demonstrate that SARS-CoV-2 N can act as a potent IFN antagonist during viral infection, and propose a novel mechanism underlying this IFN antagonizing function (Fig. 1m).

In summary, our study uncovers for the first time the mechanism by which SARS-CoV-2 evades IFN response, and demonstrates N protein as the SARS-CoV-2-encoded IFN antagonist, which may represent a promising target for antiviral intervention. Our findings extend our knowledge about the virus-host interactions of SARS-CoV-2, and should help better understand the pathogenesis of COVID-19.

## Supplementary information

Supplementary Information

## Supplementary information

**Supplementary Information** accompanies the paper at (10.1038/s41421-020-00208-3).

## References

[1] WHO. Coronavirus disease (COVID-19) Weekly Epidemiological Update. *Coronavirus disease (COVID-2019) situation reports.* https://www.who.int/docs/default-source/coronaviruse/situation-reports/20200824-weekly-epi-update.pdf?sfvrsn=806986d1_4. (2020).

[2] Platanias LC (2005). Mechanisms of type-I- and type-II-interferon-mediated signalling. Nat. Rev. Immunol..

[3] Hu Y (2017). The Severe Acute Respiratory Syndrome Coronavirus nucleocapsid inhibits type I interferon production by interfering with TRIM25-mediated RIG-I Ubiquitination. J. Virol..

[4] McBride R, Zyl M, Fielding B (2014). The coronavirus nucleocapsid is a multifunctional protein. Viruses.

[5] Mu J (2020). SARS-CoV-2-encoded nucleocapsid protein acts as a viral suppressor of RNA interference in cells. Sci China Life Sci..

