# Supplementary material for: SARS-CoV-2 N protein antagonizes type I interferon signaling by suppressing phosphorylation and nuclear translocation of STAT1 and STAT2
Source: Cell Discov. 2020 Sep 15;6:65. doi: 10.1038/s41421-020-00208-3 (PMC7490572; doi:10.1038/s41421-020-00208-3)
Supplement: Supplementary file 1 — Supplementary Information [file 41421_2020_208_MOESM1_ESM.pdf]

Supplementary Information for

SARS-CoV-2 N protein antagonizes type I interferon signaling by suppressing phosphorylation and nuclear translocation of STAT1 and STAT2

Jingfang Mu[1,2], Yaohui Fang[1], Qi Yang[1], Ting Shu[1,2], An Wang[1,3], Muhan Huang[1,2], Liang Jin[5], Fei Deng[1,2], Yang Qiu[1,2*], Xi Zhou[1,2,3,4*]

*Correspondence to: zhouxi@wh.iov.cn (X.Z.); and yangqiu@wh.iov.cn (Y.Q.)

This PDF file includes:

Supplementary Materials and Methods
Supplementary Tables S1
Supplementary Figures S1
Supplementary Figures S2
Supplementary Figures S3
Supplementary Figures S4
Supplementary Figures S5

Other Supplementary Materials for this manuscript include the following:

None

Supplementary Materials and Methods

Cell culture and transfection

Human embryonic kidney 293T (293T) cells and HepG2 cells were obtained commercially from the American Type Culture Collection (Manassas, VA, USA) and maintained in modified Eagle's medium supplemented with 10% fetal bovine serum (Gibco, South America, USA), 100 U/ml penicillin, and 100 μg/ml streptomycin at 37°C in an incubator with 5% CO2. Transfection of cells was conducted using Lipofectamine 2000 or Lipofectamine 3000 (Invitrogen, Carlsbad, CA, USA) according to the manufacturer's protocol.

Plasmids construction

Construction of pRK-Flag-N (SARS-CoV-2) was described previously[1]. The plasmids of ISRE promoter luciferase reporter and plasmids of STAT1/2 with HA tagged at the N-terminus were kindly provided by Dr. Xinwen Chen (Wuhan, China).

Construction of stable expression of Flag-tagged SARS-CoV-2 N protein in 293T cells

Full-length cDNA encoding SARS-CoV-2 N was amplified from pRK-Flag-N plasmid and then was inserted into pCDH-CMV-MCS-EF1-CoGFP-T2A-puro vector to obtain Flag-tagged SARS-CoV-2 N overexpressing plasmid. pCDH-Flag-N lentivirus particles were obtained by co-transfecting pCDH-Flag-N with packaging plasmids (pMD2.G and pCMV-dR8.91) into 293T cells, we obtained Flag-N lentivirus particles.

293T cells were infected with concentrated Flag-N lentivirus particles and then were screened by puromycin to obtain stable Flag-N-overexpressed cell line. The protein expression of Flag-N was detected by western blot analysis and immunofluorescence.

Virus infection

SeV was kindly provided by Dr. Xinwen Chen. All virus was amplified and titrated by standard plaque or focus-forming assays[2]. Cells were infected with SeV (50 hemagglutination units per ml). SARS-CoV-2 was kindly provided by Dr. Zhengli Shi. HepG2 cells were infected with SARS-CoV-2 at a multiplicities of infection (MOI) of 0.1 and harvested at 24 hours post infection.

Luciferase assay

Luciferase assay was performed as previously described[3]. The dual-Luciferase Reporter Assay System (Promega) was used for luciferase assays. Briefly, cells were seeded in 24-well plates and transfected luciferase reporter and pRL-TK plasmids combined with a total of 500 ng of target plasmid or empty control plasmid for 24 hrs. Subsequently, cells were stimulated with IFNα 2α (50 ng/mL), IFN-β (50 ng/mL), or left untreated for the indicated time periods and luciferase activity was measured.

Quantitative real-time reverse transcription PCR (qRT-PCR)

The qRT-PCR were performed as described previously[4]. Total cellular RNAs were isolated using TRIzol reagent (Invitrogen) according to the manufacturer’s protocols.

The quantification of transcripts was analyzed by real-time RT-PCR with the RT reagent Kit (Takara) and TB Green premix Ex Taq II (Takara). The data were normalized to levels of cellular β-actin mRNA in each individual sample. $2^{-\Delta\Delta Ct}$ method was used to calculate relative expression changes. The ratios of gene expression relative to the control were calculated by the $\Delta\Delta C_T$ method as previously described[4]. Data represented the means and standard deviations of three independent experiments. The primers for quantitative RT-PCR are in Supplementary Table S1.

Western blotting

The Western blotting were performed as described previously[5]. Cells were washed twice in cold phosphate-buffered saline (PBS) and lysed in lysis buffer (50 mM Tris-HCl [pH 7.4], 150 mM NaCl, 1% NP40, 0.25% deoxycholate, and a protease inhibitor cocktail [6]), and the lysates were subjected to SDS-PAGE on 12% gels by western blotting according to standard procedures with the relevant antibodies. Rabbit anti-GAPDH, HA, STAT1, phospho-STAT1 (Tyr701), STAT2, phospho-STAT2 (Tyr690), antibodies were all purchased from Cell Signaling Technology. Mouse anti-Flag were from Sigma-Aldrich.

Co-Immunoprecipitation (Co-IP)

Co-IP was performed as previously described[7]. Cells were rinsed with ice-cold PBS and lysed with RIPA buffer (50 mM Tris, pH7.5, 1 mM EDTA, 1% Triton X-100, 150 mM NaCl, 2 mM DTT) and a protease inhibitor cocktail (Roche). The cell lysates were centrifuged at 12000 ×g at 4 ℃ for 10 min and the supernatants were incubated with anti-HA together with protein-A/G agarose beads (Roche) at 4 ℃ overnight according

to the manufacturer's protocol. The immunoprecipitated samples were centrifuged and washed three times and subjected to Western blots using anti-HA and anti-Flag.

Cell fractionation

Cells were rinsed with ice-cold PBS and lysed using Cell fractionation kit (Beyotime, China) according to the manufacturer's instructions. The purified cytoplasmic fraction and the nuclear fraction were subjected to Western blot assays according to standard procedures with the relevant antibodies. Histone H3 (abcam, ab1791), Tubulin (Cell Signaling Technology), phospho-STAT1 (Tyr701), and phospho-STAT2 (Tyr690) antibodies were used.

Immunofluorescence assay

The immunofluorescence assays were performed as described previously[8]. Briefly, the cells were fixed with 3.7% paraformaldehyde in PBS for 30 min, permeabilized with 0.5% Triton X-100 and blocked in 1% normal goat serum (Boster) in PBS for 30 min on ice. The cells were incubated with anti-HA (1:500 dilution, Invitrogen) or anti-Flag (1:500 dilution, Sigma) primary antibodies. The secondary antibodies were Alexa Fluor 568- or 488-conjugated anti-rabbit and anti-mouse antibodies (1:800 dilution, Invitrogen). The nuclear DNA was stained with DAPI (Beyotime).

Reference

1 Mu, J. *et al.* SARS-CoV-2-encoded nucleocapsid protein acts as a viral suppressor of RNA interference in cells. *Sci China Life Sci*, 1-4,(2020).

2 Yang, Q. *et al.* Host HDAC4 regulates the antiviral response by inhibiting the phosphorylation of IRF3. *J Mol Cell Biol* **11**, 158-169,(2019).

3 Yang, Q. *et al.* Tick-borne encephalitis virus NS4A ubiquitination antagonizes type I interferon-stimulated STAT1/2 signalling pathway. *Emerg Microbes Infect* **9**, 714-726,(2020).

4 Li, Y. *et al.* Mechanisms and Effects on HBV Replication of the Interaction between HBV Core Protein and Cellular Filamin B. *Virol Sin* **33**, 162-172,(2018).

5 Miao, M. *et al.* Proteomics Profiling of Host Cell Response via Protein Expression and

Phosphorylation upon Dengue Virus Infection. *Virologica Sinica* **34**, 549-562,(2019).
6 Hadjadj, J. *et al.* Impaired type I interferon activity and exacerbated inflammatory responses in severe Covid-19 patients. *medRxiv*, 2020.2004.2019.20068015,(2020).
7 Lu, B. *et al.* Induction of INKIT by Viral Infection Negatively Regulates Antiviral Responses through Inhibiting Phosphorylation of p65 and IRF3. *Cell Host Microbe* **22**, 86-98 e84,(2017).
8 Mu, J. *et al.* Autographa californica Multiple Nucleopolyhedrovirus Ac34 Protein Retains Cellular Actin-Related Protein 2/3 Complex in the Nucleus by Subversion of CRM1-Dependent Nuclear Export. *Plos Pathog* **12**, e1005994-e1005994,(2016).

Table S1. The primers for qRT-PCR

Name	Sequences(5′-3′)
RBD-FOR	CAATGGTTTAACAGGCACAGG
RBD-REV	CTCAAGTGTCTGTGGATCACG
ISG56-FOR	TCCCCTAAGGCAGGCTGTC
ISG56-REV	GACATGTTGGCTAGAGCTTCTTC
ISG15-FOR	GAGAGGCAGCGAACTCATCTT
ISG15-REV	CCAGCATCTTCACCGTCAGG
ISG54-FOR	GGTCTCTTCAGCATTTATTGGTG
ISG54-REV	TGCCGTAGGCTGCTCTCCA
β-actin-FOR	AGAGCTACGAGCTGCCTGAC
β-actin-REV	AGCACTGTGTTGGCGTACAG

Supplementary Fig. S1

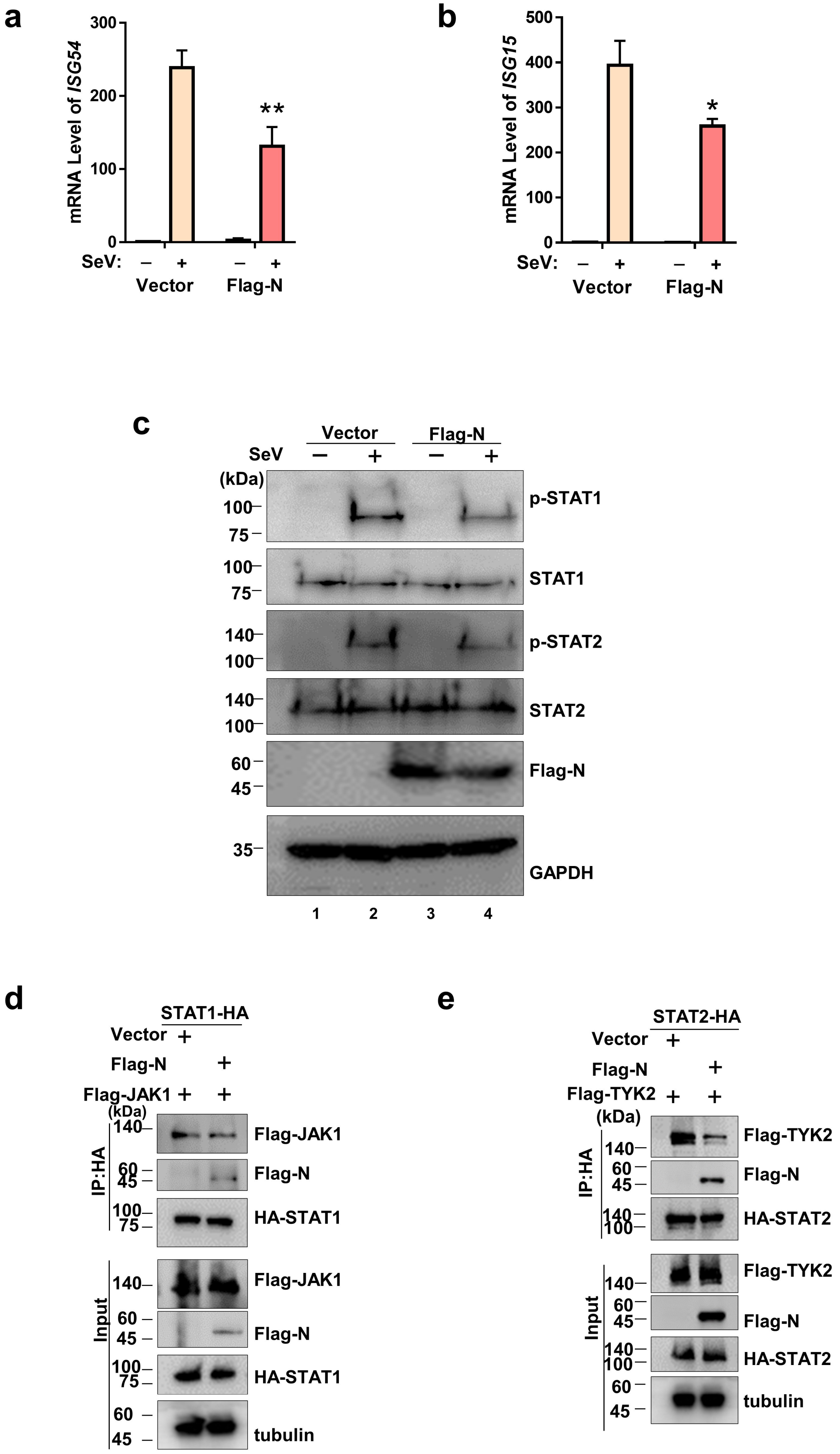

Supplementary Fig. S1 a-b 293T cells were co-transfected with plasmids with either empty vector or the plasmid encoding Flag-N (0.5μg each), as indicated. At 24 hpt, cells were then infected with or without SeV, Total RNAs were extracted at 12 hpi. The mRNA levels of ISG54 and ISG15 were measured by real-time RT-PCR analysis, and normalized to the cellular β-actin mRNA. Student's t-test was used for estimation of statistical significance. *, $P < 0.05$ and **, $P < 0.01$. Data were from three independent experiments. Mean values and standard deviations from three independent experiments per group. **c** we constructed 293T cell lines stably expressing Flag tagged SARS-CoV-2 N protein. Cells were seeded on 6-well plate and then infected with or without SeV for 12 h, The STAT1 and STAT2 protein levels and their phosphorylation levels were detected by Western blot. The concentration of total protein was quantified using the BCA method. GAPDH serves as the loading control. **d-e** 293T cells were transfected with plasmids encoding STAT1-HA and Flag-JAK1 (**d**), or plasmid encoding STAT2-HA and FLAG-TYK2 (**e**), together with either empty vector or the plasmid encoding Flag-N. At 48 hpt, the cells were harvested and subjected to a Co-IP assay using anti-HA. The immunoprecipitated proteins and the inputs were separated by SDS-PAGE and probed with anti-HA and anti-Flag.

Supplementary Fig. S2

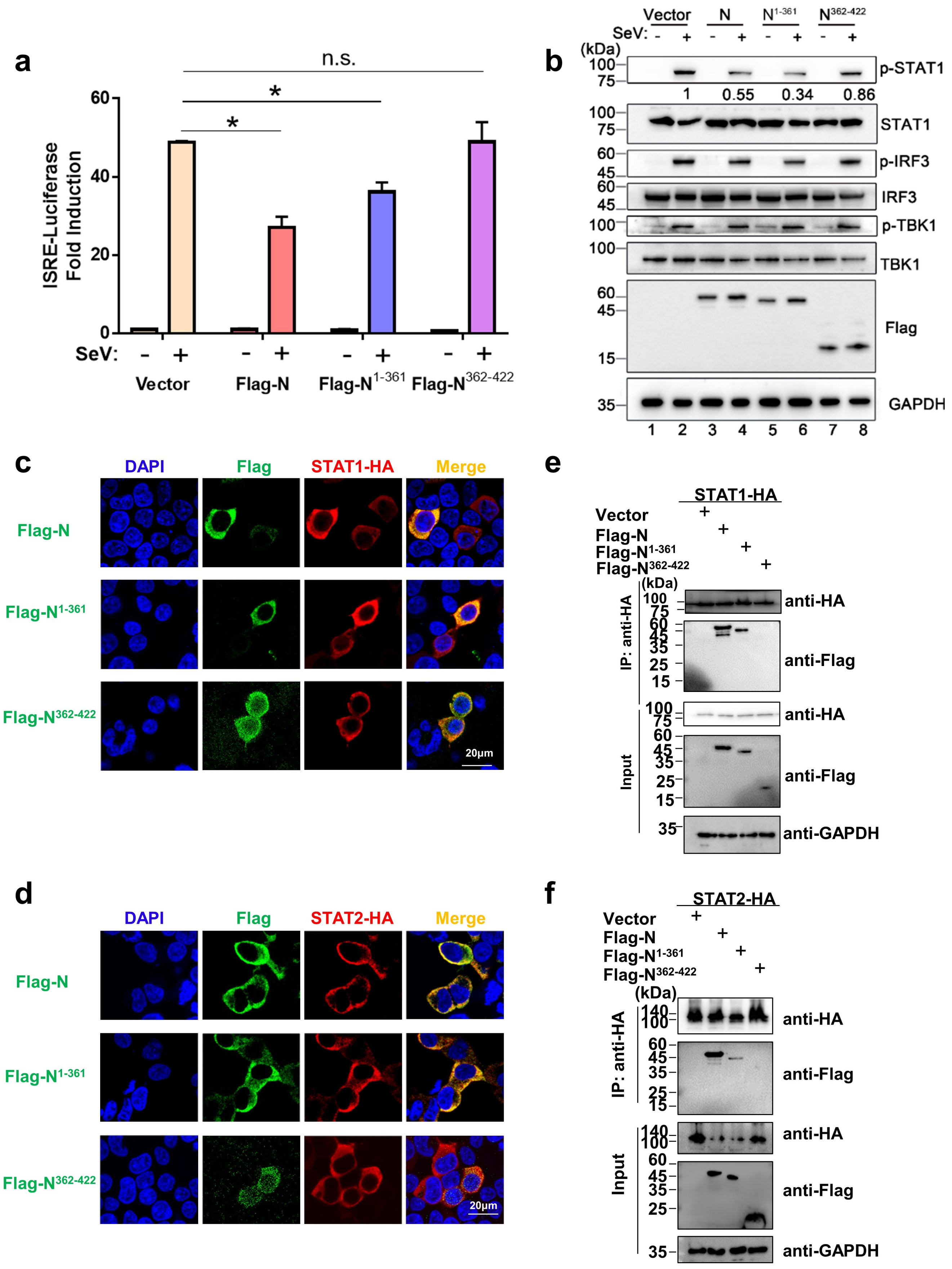

Supplementary Fig. S2 The N-terminal domain of SARS-CoV-2 N protein is sufficient for antagonizing IFN signaling. **a** 293T cells were co-transfected with plasmids with ISRE-Luc and pRL-TK, together with either empty vector or the plasmid encoding Flag-N, Flag-N^{1-361}, Flag-$N^{362-422}$ (0.5μg each), as indicated. At 24 hpt, cells were then infected with or without SeV, at 12 hpi, the reporter activity was detected by dual luciferase reporter system. Student's *t*-test was used for estimation of statistical significance. n.s., there was no significant difference, *, $P < 0.05$. Data are from three independent experiments. Mean values and standard deviations from three independent experiments per group. **b** 293T cells were transfected with either empty vector or the plasmid encoding Flag-N, Flag-N^{1-361}, Flag-$N^{362-422}$ (0.5μg each), as indicated. At 24 hpt, cells were then infected with or without SeV. At 12 hpi, Cell lysates were harvested and analyzed by western blotting with antibodies as indicated. The densities of blots were analyzed by using ImageJ software. **c-d** 293T cells were transfected with plasmids Flag-N, Flag-N^{1-361}, or Flag-$N^{362-422}$, together with the plasmid encoding STAT1-HA (**c**) or the plasmid encoding STAT2-HA (**d**). At 24 hpt, the cells were fixed and subjected to immunofluorescence staining assays. Scale bar, 20μm. **e-f** 293T cells were transfected with plasmids encoding STAT1-HA (**e**), or plasmid encoding STAT2-HA (**f**), together with either empty vector or the plasmid encoding Flag-N, Flag-N^{1-361}, or Flag-$N^{362-422}$, At 48 hpt, the cells were harvested and subjected to a Co-IP assay using anti-HA. The immunoprecipitated proteins and the inputs were separated by SDS-PAGE and probed with anti-HA and anti-Flag.

Supplementary Fig. S3

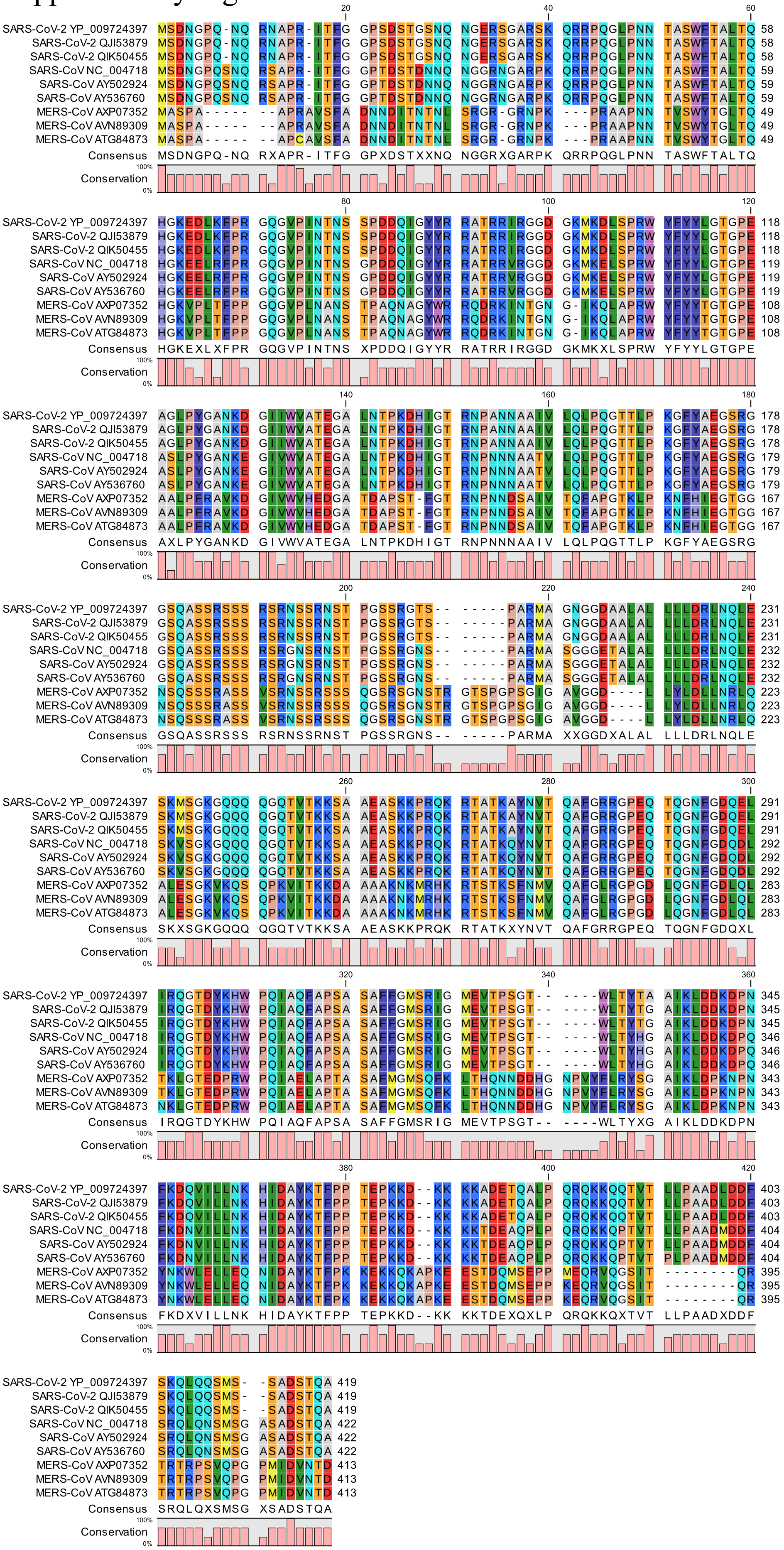

Supplementary Fig. S3 The amino acid sequence alignment of coronavirus N proteins was performed using Clustal Omega.

Supplementary Fig. S4

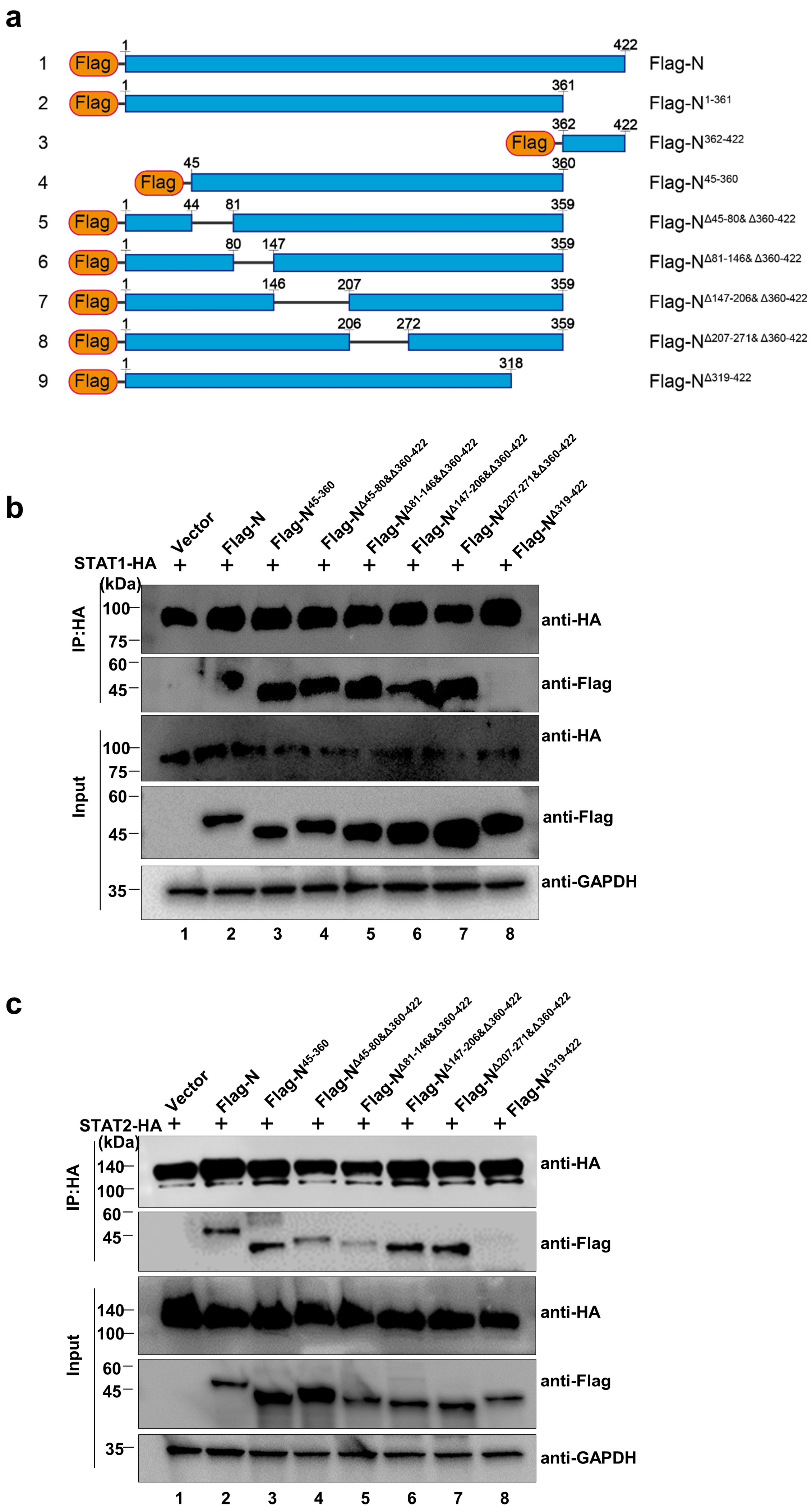

Supplementary Fig. S4 C-terminal 319-422 amino acids of SARS-CoV-2 N protein is indispensable for its binding to STAT1 and STAT2 **a** Schematic diagram of N protein and N truncations. **b-c** 293T cells were transfected with plasmids empty vector, Flag-N, Flag-N$^{45\text{-}360}$, Flag-N$^{\Delta 45\text{-}80\& \ \Delta 360\text{-}422}$, Flag-N$^{\Delta 81\text{-}146\& \ \Delta 360\text{-}422}$, Flag-N$^{\Delta 147\text{-}206\& \ \Delta 360\text{-}422}$, Flag-N$^{\Delta 207\text{-}271\& \ \Delta 360\text{-}422}$, or Flag-N$^{319\text{-}422}$, together with the plasmid encoding STAT1-HA (b) or the plasmid encoding STAT2-HA (**c**). At 48 hpt, the cells were harvested and subjected to a Co-IP assay using anti-HA. The immunoprecipitated proteins and the inputs were separated by SDS-PAGE and probed with anti-HA and anti-Flag.

Supplementary Fig. S5

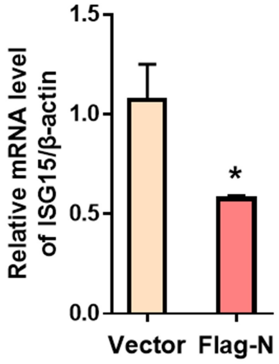

Supplementary Fig. S5 Huh7 cells were transfected with either empty vector or the plasmid encoding Flag-N. At 24 hpt, cells were infected with SARS-CoV-2 at a MOI of 0.1. At 24 hpi, total RNAs were extracted and the levels of ISG15 were measured by real-time RT-PCR analysis, normalized to the cellular β-actin mRNA. Mean values and standard deviations from three independent experiments are shown. Student's t-test was used for estimation of statistical significance. *, $P < 0.05$.
